# An Integrated Approach Based on Swarm Decomposition, Morphology Envelope Dispersion Entropy, and Random Forest for Multi-Fault Recognition of Rolling Bearing

**DOI:** 10.3390/e21040354

**Published:** 2019-04-01

**Authors:** Shuting Wan, Bo Peng

**Affiliations:** Department of Mechanical Engineering, North China Electric Power University, Baoding 071000, China

**Keywords:** swarm decomposition, morphology envelope dispersion entropy, random forest, multi-fault recognition, rolling bearing

## Abstract

Aiming at the problem that the weak faults of rolling bearing are difficult to recognize accurately, an approach on the basis of swarm decomposition (SWD), morphology envelope dispersion entropy (MEDE), and random forest (RF) is proposed to realize effective detection and intelligent recognition of weak faults in rolling bearings. The proposed approach is based on the idea of signal denoising, feature extraction and pattern classification. Firstly, the raw signal is divided into a group of oscillatory components through SWD algorithm. The first component has the richest fault information and perceived as the principal oscillatory component (POC). Secondly, the MEDE value of the POC is calculated and used to describe the characteristics of signal. Ultimately, the obtained MEDE values of various states are trained and recognized by being input as the feature vectors into the RF classifier to achieve the automatic identification of rolling bearing fault under different operation states. The dataset of Case Western Reserve University is conducted, the proposed approach achieves recognition accuracy rate of 100%. In summary, the proposed approach is efficient and robust, which can be used as a supplement to the rolling bearing fault diagnosis methods.

## 1. Introduction

Rolling bearings are the basic parts in the industrial fields, the running status of which indirectly affects the production and life safety [1,2,3]. Vibration signal analysis method, mainly composed of fault feature extraction and pattern classification, is a widely used approach for state detection and fault diagnosis [4,5]. However, in a practical engineering applications environment, complex vibration transmission paths and serious environmental noise disturbances cause it to be relatively hard to recognize the characteristic contained in the signal of a slightly damaged rolling bearing. Therefore, it is of enormous importance to develop a diagnosis approach that can realize effective detection and intelligent recognition of weak faults in rolling bearings.

In recent years, with the development of entropy theory, the nonlinear dynamic indexes, including Shannon entropy [6], approximate entropy (ApEn) [7], sample entropy (SampEn) [8], permutation entropy (PE) [9], and fuzzy entropy (FE) [10] are broadly applied to represent the non-linear characteristics of vibration signals, which greatly enriches the fault diagnosis technology. Wan et al. [11] used Shannon entropy to optimize the spectral kurtosis method, which has good immunity to random noise. Yan et al. [12] measured the regularity of mechanical structural signals by ApEn, and the functionality of mechanical structures is evaluated. Zheng et al. [13] put up with an approach on the basis of FE to discriminate the fault types of rolling bearing. SampEn is an improved form of ApEn, which is used by Ning et al. [14] and Zhang et al. [15] to detect rolling bearing fault and reduce the noise of gear vibration signals respectively. PE is applied to mutation detection of vibration signals, and the improved tensor-based singular spectrum algorithm based on PE has better performance on extracting and highlighting the early weak fault feature of rolling bearing [16]. These methods have good diagnostic results and broaden the diagnostic thinking, the defects of which, however, cannot be ignored. For example, SampEn has the following shortcomings: the calculation is slow, real-time is poor, and the similarity measurement is prone to mutation. The calculation of PE is fast, but the difference between the average amplitude and the amplitude is not considered. In 2016, Mostafa and Hamed [17] proposed a new irregular index, namely dispersion entropy (DE), which is fast in calculation and takes into account the relationship between the amplitudes. To a certain extent, it solved the above shortcomings of SampEn and PE. In addition, it should be noted that the demodulation operation can highlight the useful fault feature information contained in the vibration signal. Pang et al. [18] presented Teager energy entropy to reflect the periodicity of signals. In comparison, the demodulation operation based on morphology transform [19,20,21,22] has the characteristic of simplicity, fast calculation speed and low demodulation error. Thus, the concept of morphology envelope dispersion entropy (MEDE) is defined in this paper to recognize the bearing condition, which can better detect the randomness and dynamic mutation of signal.

Because of the vibration superposition of rolling bearing parts and the existence of environmental noise, the vibration signals collected by sensors show non-stationary multi-component characteristics. This reason caused that the MEDE of the original vibration signal cannot accurately represent the actual operation status of rolling bearings. Thus, preprocessing the raw signal is conducive to the follow-up analysis process.

The single time domain or single frequency domain analysis approach are hard to get comprehensive and reliable feature information. Time-frequency analysis technology that is capable of identify the frequency components and reveal their time-varying characteristics, is considered to be an effective tool for extracting mechanical state information. In 1998, Huang et al. [23] proposed a famous time-frequency processing approach, empirical mode decomposition (EMD), which has obvious welfare in analyzing non-stationary signals, but also has the shortcomings of endpoint effect and mode aliasing phenomenon. After that, a series of signal decomposition methods are presented and applied in state monitoring and fault diagnosis by researchers and scholars. Zhang et al. [24] combined ensemble empirical mode decomposition (EEMD) [25] with optimized SVM to discriminate the operation states of rolling bearing. Xu et al. [26] proposed an approach on the basis of local mean decomposition (LMD) [27], energy entropy, and improved LS-SVM to investigate the multi-mode problem of rotating machinery. Feng et al. [28] applied intrinsic time-scale decomposition [29] to realize the fault diagnosis of planetary gearbox. Wang et al. [30] used variational mode decomposition (VMD) [31] to analyze the rub-impact of stator and rotor, and concluded that the performance of VMD is favorable by comparing EMD and EEMD. Pang et al. [32] accurately extracted the characteristic band energy entropy that reflects the time-frequency information with the help of singular spectrum decomposition (SSD) [33] and realized the recognition of rotor operation combining with SVM. Using permutation entropy as the evaluation index, Lv et al. [34] selected the threshold parameters and the number of components needed to be set in adaptive local iterative filtering (ALIF) [35], and then effectively extracted and highlighted the weak fault characteristics. Zhu et al. [36] presented a fault diagnosis approach combining ALIF, MFE, and SVM, which can effectively identify various fault type and severity. Although the above decomposition approach are used to diagnose mechanical equipment fault successfully, there are still some problems that need to be improved, e.g., false component appearing, great computational complexity, signal over decomposition, parameter setting complex and et al. Recently, swarm decomposition (SWD) approach is presented to the public [37]. SWD is able to realize the fault feature separation under composite defective of rolling bearing [38]. The present paper introduced SWD into the field of fault identification for the first time, due to its outstanding performance in analyzing non-stationary multi-component signal than traditional methods.

Besides extracting the characteristic information contained in the signal, we also need to choose an appropriate machine learning algorithm for pattern recognition to diagnose the fault of rolling bearings. Presented by Leo, random forest (RF) algorithm [39] is a non-parametric classification algorithm driven by data, which does not need prior knowledge. RF has been effectively applied in engineering cases [40,41,42]. Compared with other classification methods, RF has the characteristic of high accuracy, fast learning speed, good anti-noise and anti-singular value. In addition, RF is not easy to appear over-fitting phenomenon and can accurately classify a large number of data. Therefore, RF is selected for fault pattern recognition in the present paper.

The present paper is aiming at developing a rolling bearing fault diagnosis approach on the basis of SWD, MEPE, and RF, which possesses high sensitivity and high fault recognition rate. The full text is composed of seven sections: Section 1 reviews the existing methods of mechanical equipment fault diagnosis. Section 2 briefly introduces SWD algorithm and illustrates its superiority by comparing it with traditional methods. Section 3 depicts the morphological transform algorithm and defines MEDE. Section 4 describes RF algorithm and its running steps. Section 5 exhibits the specific steps of the presented SWD-MEDE-RF method. Section 6 applies an open data set to verify the feasibility and superiority of the presented SWD-MEDE-RF method. Section 7 provides a few conclusions.

## 2. Swam Decomposition

The various components of signals interfere with each other and the difference of characteristics under different operation state is slight. Using SWD algorithm to process signal can not only alleviate the interference between different components, but also refine the signal to obtain more detailed characteristics.

### 2.1. Introduction of SWD Algorithm

SWD algorithm [37,38] is proposed on the basis of swarm filtering (SWF) theory, which has two basic concepts: the swarming model and the swarm-prey hunting. Some concepts are defined firstly. x[n] refers to the input discrete time series. $p_{prey}[n]$ refers to the place of the prey at *n*-step. M is the number of member in the swarm, the characteristics of each of which are described by position and velocity, i.e., $p_{i}[n]$ and $v_{i}[n]$, where *n* refers *n*-step in times. $p_{prey}[n]$, $p_{i}[n]$, and $v_{i}[n]$ are one-dimensional scalar sequence. The interaction of the driving force and the cohesion force causes the individuals of the swarm to move and hunt. The driving force is corresponding to the single member of the swarm, the equation of which is written as:

(1) $$F_{Dr-i}^{n}=P_{prey}[n]-P_{i}[n-1]$$

where, $F_{Dr-i}^{n}$ refers to the driving force of the *i*-member at *n*-step, and it is a positive force.

On the contrary, the cohesion force is corresponding to the whole member of the swarm, the equation of which is described as:

(2) $$F_{Co-i}^{n}=\frac{1}{M-1}\sum_{i=1,i\neq j}^{M}f(P_{i}[n-1]-P_{j}[n-1])$$

(3) $$f(d)=-\mathrm{sgn}(d)\ln(\frac{\left|d\right|}{d_{c}})$$

where, $F_{Co-i}^{n}$ refers to the cohesion force of the *i*-member at *n*-step in times, the attractive/repulsive effect of which is related to f(·). d refers to the length among the *i*-member and the *j*-member at *n*-step in times. $d_{r}$ is the critical distance that the two members do not interact.

The concept of the swarm–prey hunting is a simulation of bird predation. In predation, the position of each bird is constantly changing, which is written as below:

(4) $$V_{i}[n]=V_{i}[n-1]+\delta (F_{Dr-i}^{n}[n]+F_{Co-i}^{n}[n])$$

(5) $$P_{i}[n]=P_{i}[n-1]+\delta V_{i}[n]$$

where, $V_{i}[n]$ and $P_{i}[n]$ represents the position and velocity of *i*-member at *n*-step in times. The flexibility of the swarm is controlled by parameter δ.

After the predation, the positions of all the members are summed as output to SWF, and its corresponding equation is as follow:

(6) $$y[n]=\beta \cdot \sum_{i=1}^{M}P_{i}[n]$$

where, β is the scale factor that influences the sequence of M. β=0.005 affects the smallest reasonable M. The values of M and δ affect the output of SWF, the fitted curves of which corresponds to the normalized frequency W are respectively written as:

(7) $$M(w)=[33.46{\bar{w}}^{-0.735}-29.1]$$

(8) $$\delta (\bar{w})=-1.5{\bar{w}}^{2}+3.454\bar{w}-0.01$$

The core of SWD is to perform the sifting-like process iteratively. In sifting-like process, the oscillatory component (OC) with the high energy spectral density is firstly guessed, and then this component is determined by using SWF many times. In the next sifting-like process, the above determined component is firstly subtracted by the original signal, and then the OC of the remaining input signals are handled in the above same way. The sifting-like process last until no more OC is found out, i.e., the deviation of two consecutive iterations is less than the threshold $\left(T_{th}\right)$. The deviation can be calculated as:

(9) $$T=\sum \frac{{\left|y_{i}[n]-y_{i-1}[n]\right|}^{2}}{y_{i-1}{\left[n\right]}^{2}}$$

In order to run the SWD algorithm efficiently, the Savitzky–Golay (SG) filter is introduced to smooth the spectrum. In addition, a proper threshold $\left(P_{sth}\right)$ is set in advance to reduce the search scope of frequency in the process of peak selection. $w_{m}$ is the optimal frequency to be determined, and its calculation formulas are as follows:

(10) $$w_{m}=\arg\max({S^{\prime }}_{x_{it}}(w)>PS_{th})$$

(11) $${S^{\prime }}_{x_{it}}(w)=\mathrm{SG}\;filter(S_{x_{it}}(w))$$

where, $S_{x_{it}}(\cdot )$ refers to the FFT of $x_{it}[n]$.

The value of $P_{sth}$ measures around 0.1, and it determines the number of components obtained by SWD. The greater value of $P_{sth}$, the less components decomposed by SWD, and vice versa. The algorithmic framework of SWD is listed in Algorithm 1.

**Algorithm 1.** The running procedure of the SWD1: The raw signal x[n] and the threshold parameters $P_{sth}$and $T_{th}$ is initialized2: x[n] is discretized into$x_{it}[n]$, and *it* is the number of discretizations. $x_{it}[n]$ is assigned to $y_{i}[n]$, *i* = 0.3: The optimal frequency band of $y_{i}[n]$ is calculated through Equations (10) and (11).4: The parameters M and δ of SWF is calculated through Equations (7) and (8).5: The output of SWF is calculated through Equation (6), which is assigned to $y_{i}[n]$, *i* = 1. 6: The iteration deviate T is calculated through Equation (9). If $T<T_{th}$, $y_{i}[n]$ is assigned to ${x^{\prime }}_{it}[n]$. Other, Step 2–6 are run repeatedly, $y_{i}[n]$ is assigned to$x_{it}[n]$, *i* = *i* + 1.7: The remaining data $x_{it+1}[n]=x_{it}[n]-{x^{\prime }}_{it}[n]$ is obtained. If ${S^{\prime }}_{x_{it}}(w)<PS_{th}$, Step 2–6 are run repeatedly, $x_{it+1}[n]$is assigned to $x_{it}[n]$.

### 2.2. Comparsion of SWD and Other Algorithms

To illustrate the decomposition performance of SWD algorithm, a multi-component signal is constructed for analysis. The multi-component is consisted of 2 AM-FM signals, the equation of which is as follows:

(12) $$x(t)=x_{1}(t)+x_{2}(t)$$

(13) $$\left\{ \begin{array}{l} x1(t)=1.5\cos(20\pi t)\sin(1200\pi t+\cos(20\pi t))\\ x2(t)=(3+3\cos(20\pi t))\sin(700\pi t+5\cos(10\pi t)) \end{array}\right.$$

where, the sampling frequency and simulation time of each signal are set to 2048 Hz and 1s respectively.

**Figure 1:**
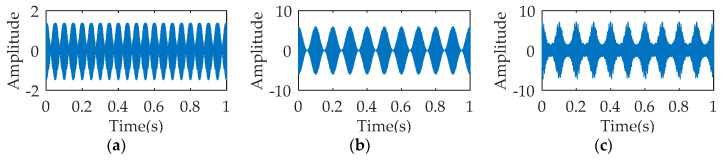
Temporal waveform: (**a**) *x*1(*t*); (**b**) *x*2(*t*); (**c**) *x*(*t*).

**Figure 2:**
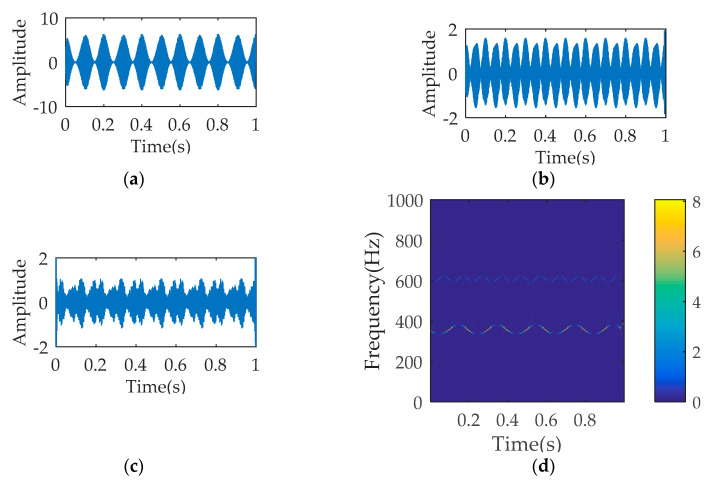
The analysis results of SWD: (**a**) temporal waveform of decomposition signal 1; (**b**) temporal waveform of decomposition signal 2; (**c**) temporal waveform of decomposition signal 3; (**d**) time-frequency diagram.

**Figure 3:**
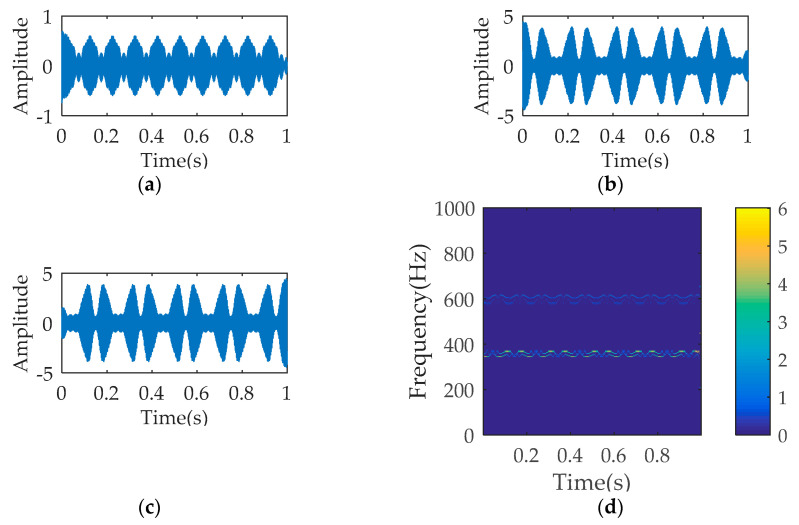
The analysis results of variational mode decomposition (VMD): (**a**) temporal waveform of decomposition signal 1; (**b**) temporal waveform of decomposition signal 2; (**c**) temporal waveform of decomposition signal 3; (**d**) time-frequency diagram.

**Figure 4:**
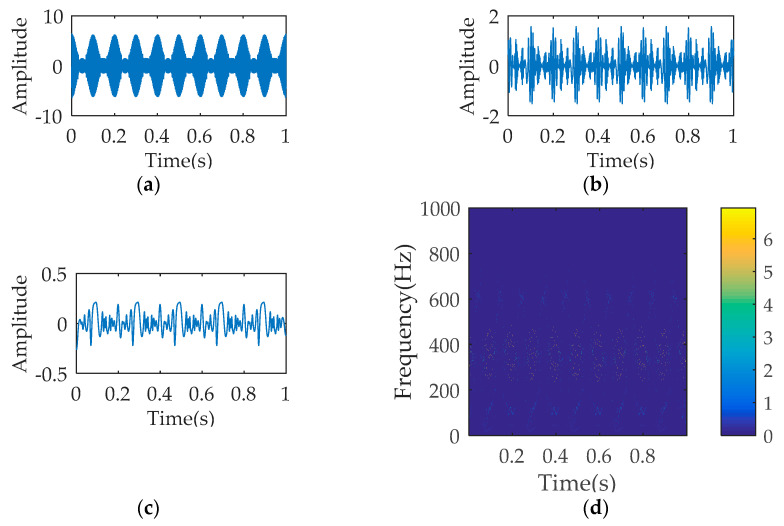
The analysis results of empirical mode decomposition (EMD): (**a**) temporal waveform of decomposition signal 1; (**b**) temporal waveform of decomposition signal 2; (**c**) temporal waveform of decomposition signal 3; (**d**) time-frequency diagram.

Figure 1 describes the temporal waveform of the single signals and the synthetic signal. The process results of SWD are illustrated in Figure 2. As seen, the decomposition signals basically restore the waveform characteristics of the raw single signal, although leading to the distortion of amplitude. The time-frequency diagram corresponding to SWD reflects the 350 Hz component and the 700 Hz component. The multi-component signal is also processed by VMD and EMD respectively. The parameters K and α of VMD equal to 4 and 1000. Figure 3a–c describes the first three decomposition signals of VMD, which cannot restore the waveform characteristics of the original single signal. In Figure 3d, the 350 Hz component and the 700 Hz component appear, but the over decomposition phenomenon also appear. The parameters of VMD are changed several times, but the decomposition results are not ideal. Figure 4 is the analysis results of EMD, where the decomposition components cannot restore the original signals, and the time-frequency diagram cannot reflect the useful frequency information either.

In conclusion, the decomposition signals obtained by SWD are simpler and do not destroy the inherent law of raw signal. Thus, using SWD to pre-process the original rolling bearing signal can be conducive to the follow-up analysis process.

## 3. Morphology Demodulation Dispersion Entropy

### 3.1. Definition of Dispersion Entropy

Dispersion entropy (DE) is utilized to evaluate the complexity and irregularity of data [17]. Setting the length of 1-dimensional (1-D) signal *x* = {*x_i_*, *i* = 0, 1, …, *N*−1} is *N*, the DE of signal *x* can be calculated by following steps:

(1) Signal x is mapped to y by the normal distribution function.
  (14) $$y_{i}=\frac{1}{\sigma \sqrt{2\pi }}\int_{-\infty }^{xi}e^{\frac{-{\left(t-\mu \right)}^{2}}{2\sigma^{2}}}dt$$
  where, *y =* {*y_i_, i* = 0, 1, …, *N*−1}, *y_i_* ∈ (0,1). *μ* and *σ*^2^ represent expectation and variance respectively.
(2) y is mapped to z by linear transformation.
  (15) $$Z^{c}=R(c\cdot y_{i}+0.5)$$
  where, $Z^{c}=\left\{Z_{i}^{c},i=1,2,\ldots ,c\right\}$. *c* represents class number.
(3) Embedded Vector $Z_{j}^{m,c}$ can be obtained as below.
  (16) $$Z_{j}^{m,c}=\left\{Z_{j}^{c},Z_{j+d}^{c},\ldots ,Z_{j+(m-1)d}^{c}\right\},\;i=1,2,\ldots ,N-(m-1)d$$
  where, *m* and *d* refer embedding dimension and time delay respectively.
(4) Dispersion pattern $\pi_{v_{0}v_{1}\ldots v_{m-1}}(v=1,2,\ldots ,c)$ is calculated. If $z_{i}^{c}=v_{0},z_{i+d}^{c}=v_{1},\ldots ,z_{i+(m-1)d}^{c}=v_{m-1}$, the dispersion pattern of $z_{i}^{m,c}$ is $\pi_{v_{0}v_{1}\ldots v_{m-1}}$. The number of dispersion pattern is $c^{m}$ for $\pi_{v_{0}v_{1}\ldots v_{m-1}}$ is composed of *c*-figures and each figure has *m* values.
(5) The probability of each dispersion pattern is calculated.
  (17) $$p(\pi_{v_{0},v_{1},\ldots ,v_{m-1}})=\frac{Number(\pi_{v_{0},v_{1},\ldots ,v_{m-1}})}{N-(m-1)d}$$
  where, A refers to the number of mappings from $Z_{j}^{m,c}$ to $\pi_{v_{0},v_{1},\ldots ,v_{m-1}}$. In other words, $p(\pi_{v_{0},v_{1},\ldots ,v_{m-1}})$ can be obtained by dividing the number of mappings from $Z_{j}^{m,c}$ to $\pi_{v_{0},v_{1},\ldots ,v_{m-1}}$ by the number of elements in $Z_{j}^{m,c}$.
(6) According to the Shannon entropy theory, the DE of 1-D signal x is defined as:
  (18) $$\mathrm{DE}(x,m,c,d)=-\sum_{\pi =1}^{c^{m}}p(\pi_{v_{0},v_{1},\ldots ,v_{m-1}})\ln(p(\pi_{v_{0},v_{1},\ldots ,v_{m-1}}))$$

### 3.2. Modified Dispersion Entropy

Morphological transformation matches the edge information of the signal through structural element probes and achieves the effective extraction of local features of the signal. When different components of rolling bearing are damaged, the edge characteristics of vibration signals are different. After vibration signals processed by morphological transformation, the disturbance information which interferes the fault type recognition are suppressed, and the impact characteristic of vibration signals are enhanced.

Corrosion, expansion, open and close transformation are the four basic operators in mathematical morphology. Setting the length of 1-D signal *f*(*n*) (*n* = 0, 1, …, *N*−1) and structure element *g*(*m*) (*m* = 0, 1, …, *M*−1) are *N* and *M* respectively, and *N* >> *M*. Using structural element *g*(*m*) to perform corrosion, expansion, open, and closed operation on signal *f*(*n*) can be defined as the following equations:

(19) $$\left\{ \begin{array}{l} \left(f\Theta g)(n)=\min[f(n+m)-g(m)\right]\\ \left(f⊕g)(n)=\max[f(n-m)+g(m)\right]\\ \left(f\;o\;g)(n)=(f\Theta g⊕g)(n\right)\\ \left(f•g)(n)=(f⊕g\Theta g)(n\right) \end{array}\right.$$

Reference [43] proposed a new morphological operator, namely average combination difference morphological filter (ACDIF), which is obtained by summing, differencing and averaging the four basic morphological operators. The equation of ACDIF is defined as:

(20) $$\mathrm{ACDIF}(f(n))=\frac{\left(f•g⊕g)(n)-(f\;o\;g\Theta g)(n)+(f⊕g•g)(n)-(f\Theta g\;o\;g)(n\right)}{2}$$

**Figure 5:**
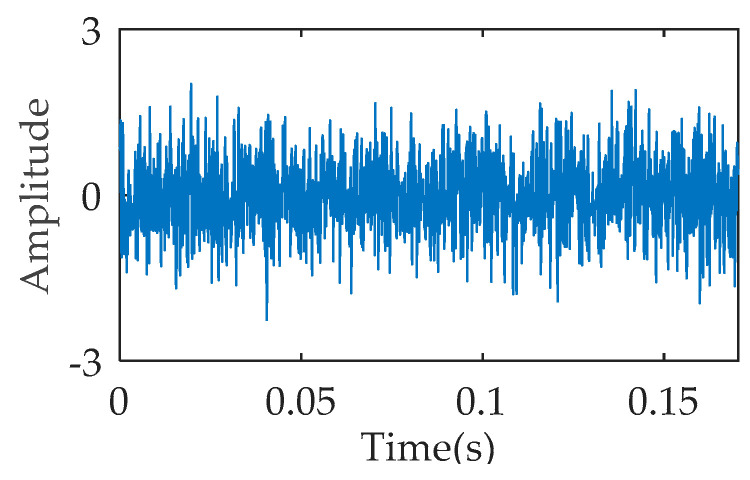
Temporal waveform of the simulation signal.

**Figure 6:**
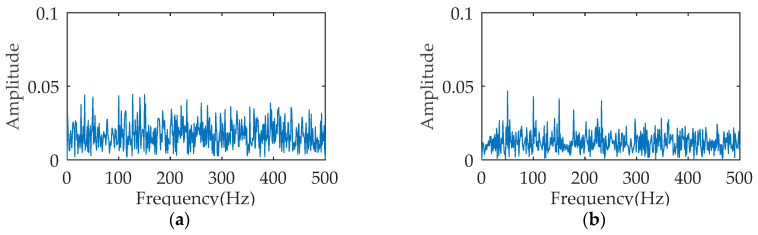
Envelope spectrum: (**a**) original signal; (**b**) filtered signal.

**Figure 7:**
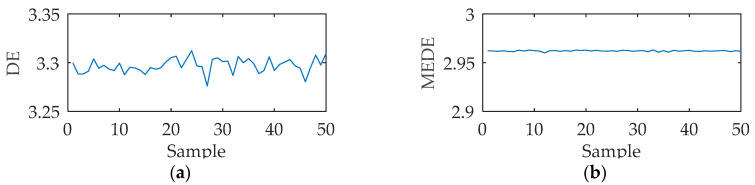
The trend of entropy change: (**a**) dispersion entropy (DE); (**b**) morphology envelope dispersion entropy (MEDE).

ACDIF transform can enhance the extraction of impact features while maintaining signal noise reduction. Figure 5 is the temporal waveform of a rolling bearing simulation signal under outer ring fault, with the impact frequency of 50 Hz. The simulation signal is processed by ACDIF transform, and Figure 6 shows its envelope spectrum before and after being processed. As seen, ACDIF transform can enhance the impact characteristics and highlight the rolling bearing vibration information. Based on this, the instantaneous envelope of ACDIF transform result is utilized as the input of dispersion entropy, and the new entropy is called morphology envelope dispersion entropy (MEDE). The superiority of the MEDE is verified by a group of signals. A total of 50 signals with the same SNR are obtained through appending −12 dB Gaussian noise to the signal shown in Figure 5 in turn. According to a recommendation by Reference [17], the embedding dimension, class number and time delay of DE is set as *m* = 3, *c* = 3, and *t* = 1 respectively in this paper. The DE and MEDE of the above 50 signals are calculated and then illustrated by a line chart as shown in Figure 7. As can be seen, the fluctuation range of MEDE is smaller than that of DE, and the calculation results of MEDE are more stable. The comparison result illustrates that the MEDE performs better in measuring signal complexity, which can effectively represent the characteristic information in the signal. 

In conclusion, the integration of MEDE and SWD can more accurately mine the intrinsic characteristic of the original data. The obtained characteristic can be utilized as the input eigenvector of the classifier to realize the identification of various operation states of rolling bearings.

## 4. RF Classifier

**Figure 8:**
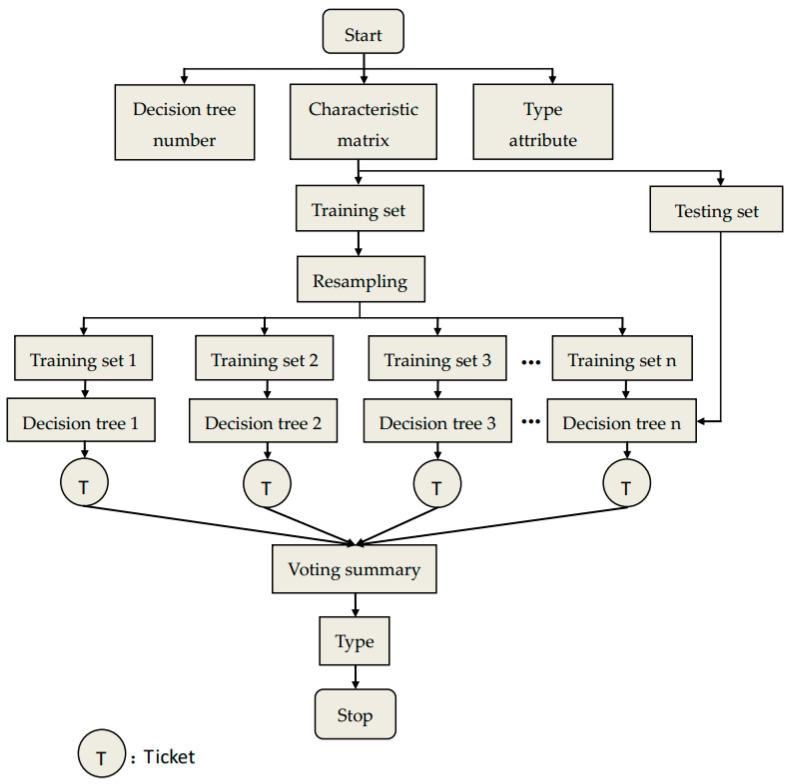
Flow chart of random forest (RF) algorithm.

Random forest (RF) is an ensemble learning model based on decision tree classifier, which contains several decision trees trained by Bagging ensemble learning technology. When inputting the samples to be classified, the final classification result is determined according to the output of a single decision tree. RF algorithm consists of the following steps, and Figure 8 is the flow chart of RF algorithm.

(1) The training sample set is selected randomly. For an original data set with n features, using Bootstrapping resampling technique, W samples are randomly selected to construct m decision trees.
(2) The split attribute set is selected randomly. For each tree node, randomly select a feature to compare and select a feature with the best classification ability to split for increasing the difference between trees and improve the generalization error.
(3) Each decision tree grows to the maximum extent without any pruning until it reached the leaf node.
(4) Form random forest. The test samples are tested by the decision tree, and the test results are determined by the majority voting of the decision tree.

RF is a non-parametric classification method driven by data, which does not need prior knowledge. Compared with other classification methods, RF has the characteristic of high accuracy, fast learning speed, good anti-noise, and anti-singular value. In addition, RF is not easy to appear over-fitting phenomenon and can accurately classify the mass data.

## 5. The Presented Fault Diagnosis Approach

Because the weak defective of rolling bearing is difficult to recognize accurately, combining the excellent characteristics of SWD, MEDE, and RF, an integrated method named SWD-MEPE-RF was developed for the present paper. This current work has three improvements as follows:

(1) Simplifying complex multi-component signals can lay a foundation for subsequent feature extraction. SWD is introduced to decompose the origin signal, which can effectively overcome the mode aliasing problem without complex parameter adjustment.
(2) Combining the advantages of dispersion entropy and morphological filtering, a feature extraction method named MEDE is proposed. MEDE can not only detect the randomness and dynamic mutation of signal, but also has good stability.
(3) Aiming at extracting fault features corresponding to weak defects from vibration signals, SWD-MEDE is proposed, which can better precisely mine the intrinsic characteristic information of signal.

**Figure 9:**
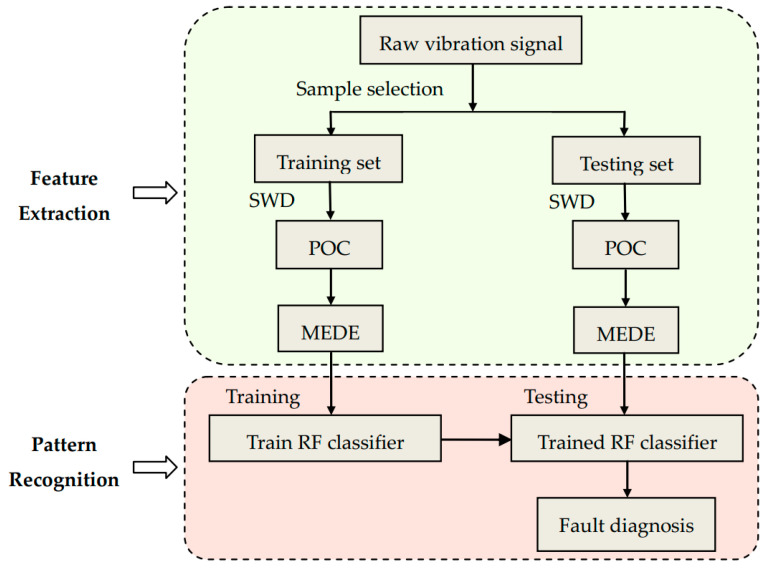
Diagnostic flow chart of the presented approach. POC: principal oscillatory component.

The specific steps of the presented method are as below, and its flow chart is illustrated in Figure 9.

(1) The raw signals are intercepted to form training set and test set. Assuming that the original signals contains *N* operation states, and the signal of each operation state is intercepted without overlapping to form *M* data samples. The sum of data samples is *Z* = *M* × *N*, *X* samples are randomly chosen as training set, and the rest *Y* = *Z* − *X* samples are used as training set.
(2) The training samples and test samples are processed by SWD algorithm. Each sample is divided into a group of oscillatory components. The first decomposed component has the richest fault information, which is named as the principal oscillatory component (POC).
(3) The MEDE of each POC are calculated and used as state characteristics.

The MEDE corresponding to training samples are the input of RF classifier, and *N* operation states of rolling bearing are the output to train the classifier. When the MEDE corresponding to the testing samples are input to the trained RF classifier, the output of which can identify the different operation states of the rolling bearing.

## 6. Application

### 6.1. Experimental Equipment and Data Collection

**Figure 10:**
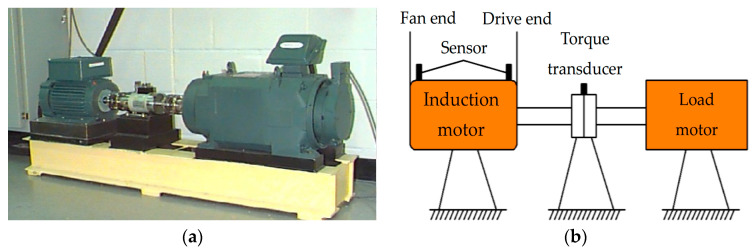
Case experimental platform: (**a**) physical photo; (**b**) sketches.

The open data set of Case Western Reserve University (CWRU) has helped many researchers to validate new technologies, theories, and technologies [44]. Figure 10a,b are the physical photo and sketches of the experimental platform of CWRU. As shown, the left part is an induction motor as the driving source, a torque transducer is arranged in the middle, and the right part is a load motor. The rolling bearing supporting the spindle is the tested object, and its surface of inner ring, outer ring, and rolling element has pitting defective processed by electric discharge machining technology, respectively. The pitting diameters of each position are 0.007, 0.014, 0.021, and 0.028 inches respectively.

**Figure 11:**
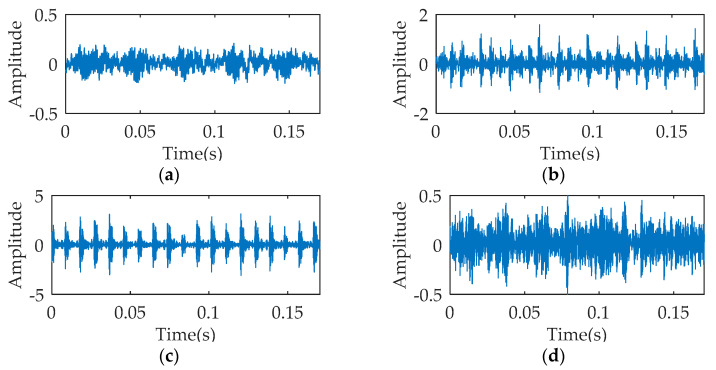
Temporal waveform of one data sample under four operation states: (**a**) normal (NOR); (**b**) inner ring fault (IRF); (**c**) outer ring fault (ORF); and (**d**) rolling element fault (REF).

For verifying the diagnostic performance of slight faults, the data of fan end rolling bearings with 0.007 inches fault diameter are selected. In the data acquisition process, the spindle speed, sampling frequency, and motor load are set to 1797 rpm, 12,000 Hz, and 0 HP respectively. The data set contains data in four operation states, i.e., normal (NOR), inner ring fault (IRF), outer ring fault (ORF) and rolling element fault (REF), and each state contains 40 samples, which are measured 2048 points in length and are intercepted from the original data. Figure 11 shows the temporal waveform of signals under four operation states. Table 1 describes the specific settings of training set and testing set. The training samples are constructed at random. The rolling bearing fault diagnosis is equivalent to a four-classification problem.

### 6.2. Analysis Results

**Figure 12:**
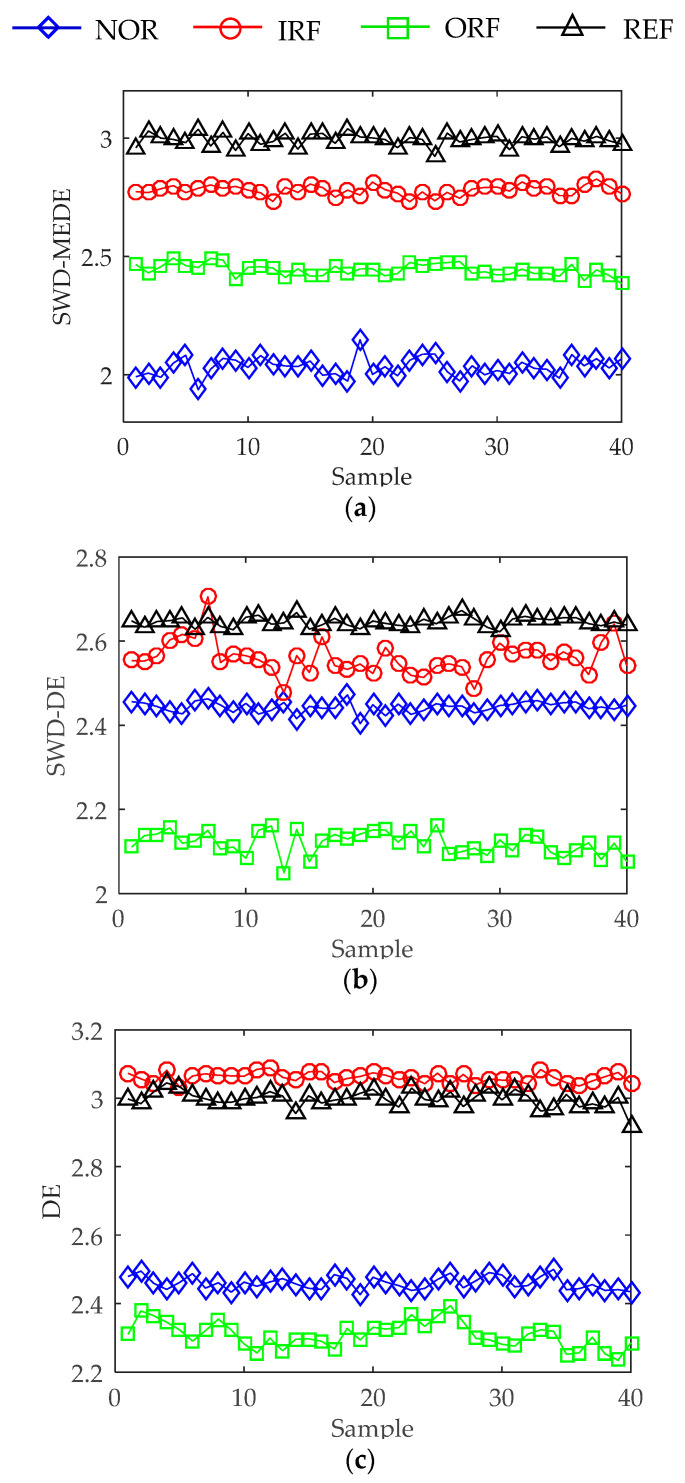
Feature extraction results: (**a**) SWD-MEDE; (**b**) SWD-DE; (**c**) DE.

According to the identification method proposed in Section 5, the vibration signals of each sample are decomposed by SWD, and the PSCs are selected to calculate their MEDE. Figure 12a illustrates the results, where the MEDE of rolling bearing signals under four operate states can be well distinguished and have good stability. The DE method (i.e., the DE of raw sample data is calculated directly), the SWD-DE method (i.e., the raw sample data is firstly decomposed by SWD, and then the DE of POC is calculated) also processed the data samples, with the results of feature extraction described in Figure 12b,c. The features under normal state and outer ring fault can be clearly separated, while the features under other two fault types have some shortcomings such as overlapping and being hardly distinguished. Compared to the SWD-DE approach, the DE approach is less effective in extracting features. In conclusion, the SWD-MEDE approach has excellent performance on feature extraction, and the extracted features have a great degree of discrimination that is able to recognize different operation states.

**Figure 13:**
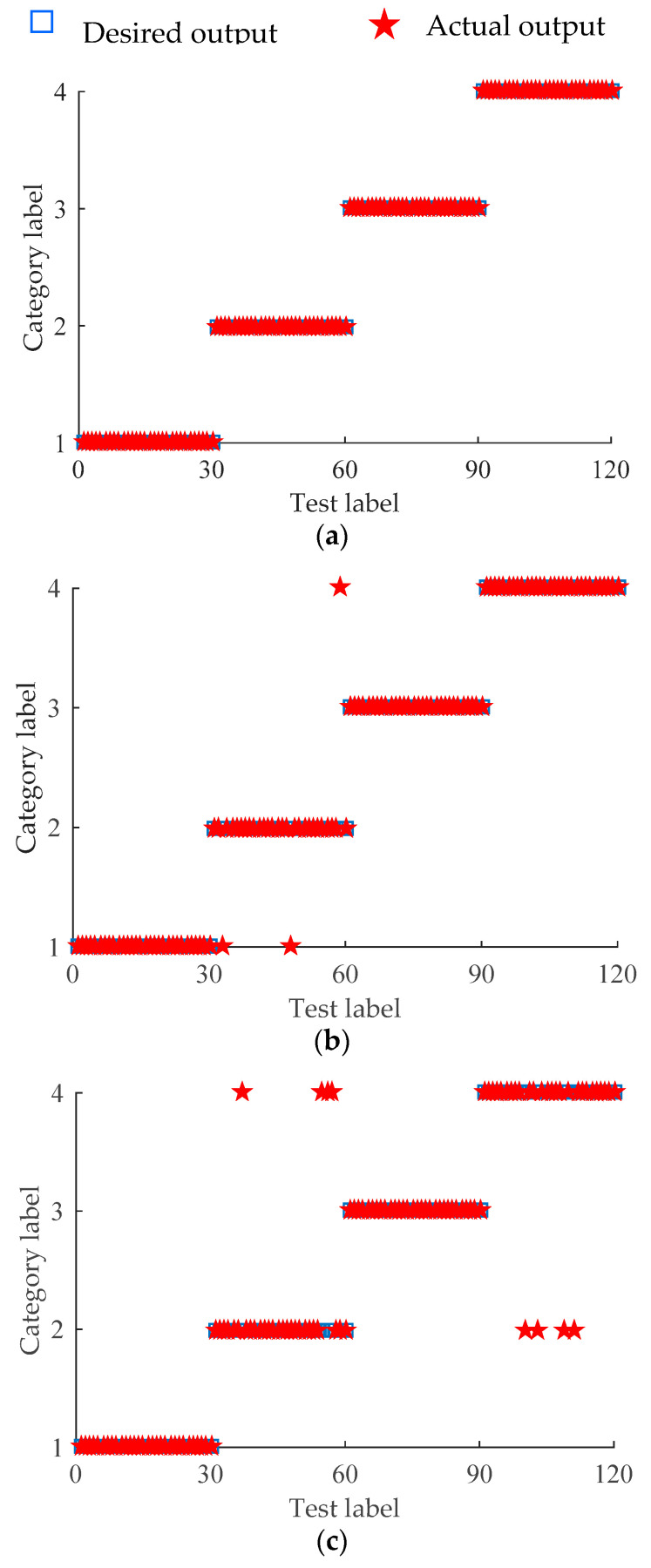
Recognition results: (**a**) SWD-MEDE-RF; (**b**) SWD-DE-RF; (**c**) DE-RF.

The features extracted by different methods are input into the RF classifier for state recognition, and Figure 13 depicts its results. Figure 13a is the classification result of the SWD-MEDE-RF method, where none of the samples are misclassified. Figure 13b is the recognition result of the SWD-DE-RF method, where the samples corresponding to NOR, ORF, and REF state are all classified correctly. Three samples corresponding to inner ring fault state are classified incorrectly, where two samples are classified as normal state, and one sample is classified as rolling element fault. Figure 13c is the recognition result of the DE-RF method, where the samples corresponding to normal and outer ring fault state are all classified correctly, four samples corresponding to inner ring fault are classified as ball fault, and four samples corresponding to ball fault are classified as inner ring fault. 

In addition, the VMD-MEDE-RF method and the EMD-MEDE-RF method are also applied to analyze the date set. Unlike the SWD-MEDE-RF method, the two methods use EMD and VMD to decompose the data and then reconstruct the sub-components according to the maximum correlation coefficient-kurtosis criterion. The MEDE of reconstructed signal is input into the classifier as feature information for state recognition. The parameters K and α of VMD are set to 4 and 1000 respectively. Table 2 lists the recognition accuracy of the mentioned approach. The average recognition accuracy of the DE-RF method, the SWD-DE-RF method, the SWD-MEDE-RF method, the VMD-MEDE-RF method and the EMD-MEDE-RF method are. 93.5%, 97.5%, 100%, 87.75%, and 69.25%, respectively. The recognition accuracy of VMD-MEDE-RF method and EMD-MEDE-RF method is not ideal and cannot meet the requirements. By analyzing the characteristics of EMD and VMD, the reason for this phenomenon is that these two decomposition methods destroy the inherent law of the original signal.

### 6.3. Futher Discussions

The rolling bearing data corresponding to the motor loads of 0, 1, 2, and 3 HP are analyzed by the proposed SWD-MEDE-RF method. The average recognition accuracy of different motor loads are all 100%. The dataset with 1024 data length are also conducted by the proposed SWD-MEDE-RF method. The average recognition accuracy is 100% too. In order to explain the recognition effect of the proposed method comprehensively, Table 3 summarizes the comparative study between the current work and the published references. The comparative items include the class number, the signal processing method, the extracted feature, the number of features, the selected classifier, and the recognition accuracy. As shown in Table 3, the proposed method requires a small number of features to achieve the classification work, and the classifier does not need special optimization. The proposed method can achieve 100% recognition accuracy for multi-class recognition of rolling bearing.

## 7. Conclusions

This present paper proposed an integrated approach, named SWD-MEDE-RF, for multi-fault recognition of rolling bearing. The proposed method is based on the idea of signal denoising, feature extraction, and pattern classification. The analysis results of CRWU dataset demonstrate that the feature information of four operate states extracted by SWD-MEDE approach can be well distinguished and have good stability, and SWD-MEDE-RF approach achieves 100% recognition accuracy for the four rolling bearing running operations. 

Compared with the published papers, this current work has three highlights. (1) SWD can analyze signal better without breaking the inherent law of signal, and there is no complicated parameter adjustment. (2) MEDE can not only detect the randomness and dynamic mutation of signal, but also has good stability. (3) The integration of SWD and MEDE can better precisely dig the intrinsic characteristic information of signal. In summary, the proposed approach can efficiently detect the weak fault feature information and accurately recognize the fault type at the beginning of rolling bearing defect. This current work can be used as a supplement to the rolling bearing fault diagnosis method.

The proposed method belongs to supervised classification that needs transcendental knowledge. In future research work, how to propose an approach on the basis of unsupervised classification is our concern. Additionally, fault degree detection and equipment parts life prediction are also our research interest.

## Figures and Tables

**Table 1:** Description of the training samples and samples.

Category Label	Operation State	Defective Diameter (inches)	Number of Training Samples	Number of Testing Samples
1	NOR	0	10	30
2	IRF	0.0007	10	30
3	ORF	0.0007	10	30
4	REF	0.0007	10	30

**Table 2:** Recognition accuracy of different operation states.

	NOR	IRF	ORF	REF
DE-RF	100%	87%	100%	87%
SWD-DE-RF	100%	90%	100%	100%
SWD-MEDE-RF	100%	100%	100%	100%
VMD-MEDE-RF	90%	87%	87%	87%
EMD-MEDE-RF	43%	97%	90%	47%

**Table 3:** Comparative analysis between the published method and the proposed method. WPD: wavelet packet decomposition; EMD: empirical mode decomposition; EEMD: ensemble empirical mode decomposition; LMD: local mean decomposition; PE: permutation entropy; MPE: multi-scale permutation entropy; IMF: intrinsic mode function; ANN: artificial neural network; SVM: support vector machine; HMM: hidden markov model; IPSO-LSSVM: least squares support vector machine optimized by improved particle swarm optimization; ICDSVM: support vector machines optimized by inter-cluster distance.

Reference	Class Number	Signal Processing Method	Extracted Feature	Feature Number	Classifier	Recognition Accuracy
[45]	4	Wavelet	PE	11	ANN/SVM	97.5%
[46]	4	WPD	MPE	8	HMM	94.2%
[47]	3	EMD	IMF energy entropy	6	IPSO-LSSVM	97%
[48]	3	EEMD	IMF-PE	5	ICDSVM	97.5%
[49]	4	LMD	MPE	14	HMM	95%
This work	4	SWD	MEDE	1	RF	100%
